# Fecal Microbiota, Bile Acids, Sterols, and Fatty Acids in Dogs with Chronic Enteropathy Fed a Home-Cooked Diet Supplemented with Coconut Oil

**DOI:** 10.3390/ani13030502

**Published:** 2023-01-31

**Authors:** Carla Giuditta Vecchiato, Carlo Pinna, Chi-Hsuan Sung, Francesca Borrelli De Andreis, Jan S. Suchodolski, Rachel Pilla, Costanza Delsante, Federica Sportelli, Ludovica Maria Eugenia Mammi, Marco Pietra, Giacomo Biagi

**Affiliations:** 1 Department of Veterinary Medical Sciences, University of Bologna, Via Tolara di Sopra 50, 40064 Ozzano Emilia, Italy; 2 Gastrointestinal Laboratory, Department of Small Animal Clinical Sciences, Texas A&M University, College Station, TX 77843, USA

**Keywords:** medium-chain fatty acids, medium-chain triglycerides, coconut oil, canine chronic enteropathies, dog fecal microbiota, fecal fatty acids, fecal sterols

## Abstract

**Simple Summary:**

Medium-chain triglycerides (MCT), which are lipid molecules made up of medium-chain fatty acids (MCFAs), can be rapidly absorbed through enterocytes, bypassing the lymphatics, and appear as interesting energy sources used to manage common canine gastrointestinal disorders such as chronic enteropathies (CE). Virgin coconut oil (VCO) is a vegetal fat source rich in MCT that has been shown to have antimicrobial properties when supplemented in piglet diets. This study aimed to evaluate the effects of a home-cooked diet (HCD) supplemented with VCO (HCD + VCO) on the clinical scores, fecal microbiota, and metabolomes of 18 CE dogs. All dogs responded well to the diet change, demonstrating improvements in their clinical signs. Compared to their habitual diets, HCD reduced fecal fat excretion, increased fecal moisture, and induced changes in the microbiota. HCD + VCO induced changes in their sterol and fecal fatty acid profiles but failed to exert significant effects on fecal microbiota. However, HCD + VCO increased the fecal proportions of certain MCFAs (C10:0 and C12:0), which are theoretically not expected to be found in feces. These results should be considered preliminary and should be confirmed in further studies with healthy dogs.

**Abstract:**

Medium-chain fatty acids (MCFAs) are considered to be interesting energy sources for dogs affected by chronic enteropathies (CE). This study analyzed the clinical scores, fecal microbiota, and metabolomes of 18 CE dogs fed a home-cooked diet (HCD) supplemented with virgin coconut oil (VCO), a source of MCFA, at 10% of metabolizable energy (HCD + VCO). The dogs were clinically evaluated with the Canine Chronic Enteropathy Activity Index (CCECAI) before and at the end of study. Fecal samples were collected at baseline, after 7 days of HCD, and after 30 days of HCD + VCO, for fecal score (FS) assessment, microbial analysis, and determination of bile acids (BA), sterols, and fatty acids (FA). The dogs responded positively to diet change, as shown by the CCECAI improvement (*p* = 0.001); HCD reduced fecal fat excretion and HCD + VCO improved FS (*p* < 0.001), even though an increase in fecal moisture occurred due to HCD (*p* = 0.001). HCD modified fecal FA (C6:0: +79%, C14:0: +74%, C20:0: +43%, C22:0: +58%, C24:0: +47%, C18:3n−3: +106%, C20:4n−6: +56%, and monounsaturated FA (MUFA): −23%, *p* < 0.05) and sterol profile (coprostanol: −27%, sitostanol: −86%, *p* < 0.01). VCO increased (*p* < 0.05) fecal total saturated FA (SFA: +28%, C14:0: +142%, C16:0 +21%, C22:0 +33%) and selected MCFAs (+162%; C10:0 +183%, C12:0 +600%), while reducing (*p* < 0.05) total MUFA (−29%), polyunsaturated FA (−26%), campesterol (−56%) and phyto-/zoosterols ratio (0.93:1 vs. 0.36:1). The median dysbiosis index was <0 and, together with fecal BA, was not significantly affected by HCD nor by VCO. The HCD diet increased total fecal bacteria (*p =* 0.005) and the abundance of *Fusobacterium* spp. (*p* = 0.028). This study confirmed that clinical signs, and to a lesser extent fecal microbiota and metabolome, are positively influenced by HCD in CE dogs. Moreover, it has been shown that fecal proportions of MCFA increased when MCFAs were supplemented in those dogs. The present results emphasize the need for future studies to better understand the intestinal absorptive mechanism of MCFA in dogs.

## 1. Introduction

Chronic enteropathies (CE) represent a heterogeneous group of common diseases in dogs, typically manifesting with clinical signs such as diarrhea, vomiting, anorexia, and/or weight loss [1]. Diet is known to play a crucial role not only in disease pathogenesis (representing a potential risk factor) but also in the therapeutic approach [2]. Dietary strategies based on the use of “limited-ingredient” diets or novel protein sources (or protein hydrolysates) are often effective in managing the gastrointestinal symptoms [3]. In this context, dietary medium-chain triglycerides (MCT), which are lipid molecules made up of medium-chain fatty acids (MCFAs), are proving to be interesting from a nutritional perspective. MCFAs, which represent saturated fatty acids ranging from 6 to 12 in carbon chain length and include caproic (C6:0), caprylic (C8:0), capric (C10:0), and lauric (C12:0) acids, are more soluble in water and biological liquids than long-chain fatty acids (LCFA), due to their lower molecular weight and smaller size. Consequently, MCFAs are rapidly and more efficiently absorbed through the intestinal mucosa and transported directly through the portal vein to the liver, bypassing the lymph system [4], and therefore represent a preferential substrate for β oxidation rather than for lipid storage [5,6]. Because of their unique digestive and metabolic properties related to the potential role of MCTs in lipid catabolism and the reduction in body fat mass, MCTs are used to overcome metabolic and digestive diseases such as fat malabsorption and obesity [5,7]. In particular, MCT-enriched diets are currently considered the primary treatment strategy for people with primary intestinal lymphangiectasia, and they have been proven to reduce overall mortality [8,9]. In dogs, MCT supplementation has as yet been described in two cases diagnosed with protein-losing enteropathy [10,11]. Studies on the swine model have demonstrated the potential of these lipid molecules as feed antibiotic replacers, improving the intestinal mucosal structure and the growth performance in weaning animals [12,13,14] and exhibiting interesting antibacterial effects in the pig intestinal environment. This is due to their capacity to penetrate the bacterial membrane, destroying internal structures (this effect seems to be exerted by lauric acid, in particular), with special regard to Gram-positive and potentially harmful bacteria [13,15].

Since the addition of non-esterified MCFA mixture in pig feed could have a negative impact on feed intake due to sensory properties (particularly over 8% of inclusion), to overcome this inconvenience, MCT-enriched ingredients have been evaluated in several studies, allowing dietary inclusion rates up to 15%, as reviewed by Zentek et al. [16].

In this context, virgin coconut oil (VCO) has recently received great attention from the scientific community because of its relatively high content of MCT (which represents more than 50% of its total fat matter), principally formed by lauric, caprylic, and capric acids [17,18]. VCO is believed to offer several health benefits, including antifungal, antioxidant, antibacterial, antiviral, hepatoprotective, and anti-inflammatory effects [19]. Moreover, beneficial effects on weight loss, glycemic index, and the immune system have been proposed for this vegetable oil [20]. Recently, in swine species, some studies have proposed the positive influence of dietary coconut oil supplementation both on the intestinal microbiota, supported by an increase in the rectal abundance of beneficial bacteria such as *Bifidobacterium* and *Lactobacillus* spp., when added to feed mixture at 3 g/kg [21], and on growth performance and immune function when it was used, at 2% of feed mixture, as an alternative to dietary antibiotics [22]. Regarding canine species, at present, there is no strong evidence supporting the positive effects of MCFA- or MCT-enriched ingredients, such as VCO, on dog health. Nevertheless, some investigations have reported a tendency toward a slight improvement in fat digestibility in healthy dogs receiving a diet supplemented with 5.7% of MCT (dietary total lipid composition around 13%) [23] and an increase in serum concentrations of cholesterol and fat-soluble vitamins in dogs affected by exocrine pancreatic insufficiency fed with a diet containing 35% of the total fat as MCT (dietary total lipid composition around 10–11%) [24]. To date, the mechanism of intestinal absorption and the utility of MCT dietary supplementation have not been elucidated in canine species, while some evidence exists regarding food aversion when MCTs have been supplemented at high doses (22–25% ME) in this species [25,26].

However, based on the evidence collected in pigs, MCTs may represent interesting energy sources and intestinal microbial modulators in dogs affected by CE, potentially those presenting with fat malabsorption and/or microbial dysbiosis [2].

We, therefore, hypothesized that VCO, as a source of MCT, may have a beneficial influence on the clinical scores, intestinal microbiomes, and metabolomes of CE dogs receiving a homemade limited-ingredient diet.

## 2. Materials and Methods

The study was carried out according to the Italian legislation that implemented the European Council Directive 2010/63 on the protection of animals used for scientific purposes. The experimental protocol was reviewed and approved by the Scientific Ethics Committee on Animal Experimentation of the University of Bologna (protocol number: 1162). All the dogs involved in the study were referred from the Unit of Gastroenterology at the Veterinary Teaching Hospital of the University of Bologna (Italy) between January 2019 and August 2020. Informed consent was obtained from all dog owners before the beginning of the study.

### 2.1. Animals

Privately owned dogs with a history of chronic gastrointestinal (GI) signs lasting >3 weeks were included. Compatible GI signs included diarrhea, vomiting, weight loss, decreased appetite, or some combination of these signs. Only dogs >1 year of age were enrolled. The presence of CE was suspected based on the previously established criteria, according to the literature [2,27]. In particular, a negative coprological test for intestinal parasites was confirmed for each dog in fecal samples collected during three consecutive days. Urine analysis, hematology, and serum biochemistry profile were performed to exclude other pathological conditions. Further examinations were completed at the discretion of the attending clinicians to exclude other causes of chronic GI signs. A dietary history regarding type of food the dogs received before baseline (commercial dry food vs. commercial wet food/home-cooked diet) was collected from each owner.

Dogs that had received antibiotics or other medical treatments in the last 30 days were excluded. During the study, the dogs continued living in their usual environment and were fed only with the provided dietary treatments.

### 2.2. Diets

A complete and balanced home-cooked diet (HCD) was designed for the study, using horse meat as a novel protein source. Minerals and vitamin supplementation were provided through products designed for human consumption to avoid any unrecognized proteins that are often used in veterinary supplements as palatability enhancers. The daily HCD amount for each dog was calculated based on the animal’s daily energy requirements following the metabolic body weight (BW), according to the recommendations for the maintenance of adult dogs: 110 kcal/kg BW^0.75^ [28]. Detailed instructions on how to prepare the HCD recipe and the daily amount to feed were provided to dog owners. The VCO that was used during the study was a commercial product available on the Italian market. It was included in the HCD at 10% of metabolizable energy (ME), replacing a calculated amount of sunflower oil used as a major lipid source in HCD formulation. The owners were instructed to simply add and mix VCO to the HCD to ensure its total consumption. Since dogs were fed twice a day, the daily dose of VCO was split and added equally to each of the two planned daily meals. The ingredients used to formulate HCD and HCD + VCO and the chemical composition of the experimental diets are reported in Table 1. Before the beginning of the study, the two experimental diets were recreated in the laboratory and, together with VCO, their acidic composition was assessed (Table 2).

### 2.3. Study Design, Clinical Evaluations, and Blood and Fecal Sample Collection

At the first physical examination (baseline), each dog’s BW was recorded, and blood samples were collected. Moreover, some relevant clinical score indices were calculated: the Canine Chronic Enteropathy Activity Index (CCECAI), according to Allenspach et al. [27]; the fecal score (FS), according to the 7-point Purina Fecal Scoring Chart; and the body condition score (BCS), following the 9-point scale proposed by Laflamme [29].

To better evaluate the differences in FS according to dog size, the animals were grouped into small breeds (≤15 kg BW) and large breeds (>15 kg BW).

Following the first visit, the HCD was gradually introduced for 4 days and then fed to dogs as the only diet for 7 days. Then, FS was checked, and from Day 8, the VCO supplementation was introduced for the following 30 days (HCD + VCO). At the end of the study, the dogs were clinically re-evaluated through CCECAI, FS, and BCS score assessments, and a blood sample was collected from each dog. Moreover, at baseline, after 7 days of dietary treatment with HCD, and then after 30 days with HCD + VCO, owners were asked to collect a sample of fresh feces. Fecal samples were frozen within 20 min of evacuation, stored at −20 °C, and successively freeze-dried until chemical (fatty acids [FA], sterols, and bile acids [BA]) and microbial analyses were performed.

### 2.4. Chemical Analyses

Blood samples were collected after 15 h of fasting, and serum chemistry parameters (albumin, total protein, cholesterol, and triglycerides) were carried out on an automated chemistry analyzer (AU480, Beckman Coulter/Olympus, Brea, CA, USA), while serum cobalamin concentration was assessed by Immulite 2000 chemiluminescent immunoassay (Diagnostic Products Corporation, Los Angeles, CA, USA).

Cobalamin levels were measured only during the first visit, while serum albumin, total protein, cholesterol, and triglyceride levels were assessed before and after the treatment with HCD + VCO.

Proximate analyses of the experimental diets (HCD and HCD + VCO) were conducted according to International Standard methods (AOAC, 2000 [30]).

A lipidic component in 2 g of freeze-dried sample (experimental diets and fecal samples) was extracted using a 2:1 *v/v* chloroform–methanol mixture [31], and 15 mg of lipids were converted to FAME by acid-catalized transesterification, using 2 mL of hydrochloric acid in methanol 0.5 M and 1 mL of hexane containing nonadecanoic acid (C19:0; 1 mg/mL of hexane) as internal standard (Sigma-Aldrich, Taufkirchen, Germany).

Gas chromatographic analysis was performed using a Shimadzu GC 2025 (Shimadzu Corp., Kyoto, Japan) equipped with a flame-ionization detector and a polar fused silica high capillary column (SP-2650 FAME GC Column, 100 m, 0.25 mm i.d., 7” cage; Supelco Inc., Bellefonte, PA, USA) and with helium as the carrier gas (30 mL/min). The total FAME profile in a 1-μL sample volume at a split ratio of 1:110 was determined using the following conditions: the oven temperature was programmed at 150 °C and held for 2 min, then increased to 220 °C at 1.5 °C/min and held for 20 min. The injector and detector temperatures were set at 250 °C. FAME identification was based on a mixture of 37 Component FAME Mix (Supelco Inc., Bellefonte, PA, USA) [32]. Fatty acids were expressed as % of total fatty acids.

A targeted metabolomic approach was used to measure fecal concentrations of bile acids and sterols, according to a protocol designed for human feces, and adapted as described by Galler et al. [33]. Fatty acids and bile acids are reported as proportions of total measured fatty acids and bile acids, respectively, while sterols are expressed as µg/mg of freeze-dried feces.

### 2.5. Microbial Analyses

DNA was extracted from an aliquot of 100 mg of feces using the MoBio Power soil DNA isolation kit (MoBio Laboratories, Carlsbad, CA, USA) according to the manufacturer’s instructions. The qPCR assays were performed as reported previously in the study by AlShawaqfeh et al. [34]. The qPCR panel consisted of eight bacterial groups: total bacteria, *Faecalibacterium* spp., *Turicibacter* spp., *Escherichia coli*, *Streptococcus* spp., *Blautia* spp., *Fusobacterium* spp., and *Clostridium hiranonis*. The qPCR data were expressed as the log amount of DNA (fg) for each particular bacterial group/10 ng of isolated total DNA. The abundance of the evaluated bacterial groups was used to calculate the canine Dysbiosis Index (DI), according to a mathematical algorithm validated by AlShawaqfeh et al. [34].

### 2.6. Statistical Analysis

The assessment of data for normality was calculated by applying the D’Agostino-Pearson test. FS and chemical and bacterial abundance data obtained from feces collected at the three sampling times (baseline, HCD, and HCD + VCO) were analyzed by repeated measures ANOVA or the non-parametric analog (Friedman test). Either Tukey’s or Dunn’s test was used for multiple comparisons. Data on fecal moisture were analyzed, taking into account the type of habitual diet (dry vs. wet food) and the dog size (lower or higher than 15 kg BW). BW, CCECAI, BCS, and selected blood parameters recorded before and after the complete dietary trial (baseline vs. HCD + VCO) were evaluated using the Wilcoxon matched pairs signed rank test or the paired *t*-test. All data, except for dietary treatments, are reported as median and range. For all statistical analyses, significance was set at *p* < 0.05. Statistical analyses were performed using GraphPad Prism version 9.2 (GraphPad Software, San Diego, CA, USA).

## 3. Results

### 3.1. Animals

Based on the inclusion criteria previously described, a total of 18 privately owned CE dogs were enrolled in the study. The sample included both pure breeds (n = 11) and mixed breeds (n = 7) dogs, with a median age of 58 months (14–120 months). Thirteen dogs were male (six castrated and seven intact) and five were spayed females. At the beginning of the study, their median BW was 16 kg (5.3–40 kg). All of the dogs presented chronic GI signs (>20 days) such as vomiting (13/18) and/or diarrhea (14/18). The dogs were fed commercial dry (11/18) or commercial wet food/home-cooked (7/18) diets before being enrolled in the study, and they had never received horse meat as a protein source. As reported by the owners, all animals showed a good acceptance of HCD. Similarly, VCO supplemented with HCD did not lead to a decrease in diet palatability or a worsening of the dogs’ health conditions.

Clinical scores and selected serum parameters, assessed at baseline and after HCD + VCO, are summarized in Table 3. CCECAI was reduced after the treatment with HCD + VCO, with median values of 3 (1–10) vs. 1 (0–6) at baseline and at the end of the study, respectively (*p* = 0.001). Conversely, BW and BCS did not show any significant changes (*p* > 0.05, Table 3).

Of all dogs, 5/18 had a cobalamin value slightly above the lower reference value (between 250 and 300 ng/L), while 6/18 presented with baseline serum cobalamin levels lower than the minimum reference value (250 ng/L). Those dogs were treated with weekly subcutaneous injections of 50 μg/kg cyanocobalamin, according to the protocol proposed by Toresson et al. [35]. Cobalamin levels, before and after the treatment with HCD + VCO, did not show significant changes (*p* > 0.05, Table 3).

FS showed an improvement in fecal firmness during the study, since a lower fecal score indicates a more normal stool consistency, with median values of 4/7 (2–7) at baseline, 3 (2–6) after HCD, and 2/7 (2–4) after treatment with HCD + VCO. A significant difference in FS was found between the baseline and HCD + VCO (*p* < 0.001).

### 3.2. Fecal Chemical Parameters

Results concerning fecal moisture, fat content, and fatty acid concentrations are reported in Table 4. Fecal moisture was higher after treatment with HCD (+8%) and HCD + VCO (+7%) compared with baseline (*p* = 0.001); on the contrary, compared with baseline, fecal fat content decreased after treatment with HCD (−26%, *p* = 0.037), but not with HCD + VCO. To achieve a better understanding of the results regarding fecal moisture, the data were analyzed by splitting the dogs into different groups according to the type of food received before baseline (commercial dry food = 11/18 vs. commercial wet food/home-cooked diet = 7/18) and dog size (small breeds = 9/18; and large breeds = 9/18). Fecal moisture was confirmed to be significantly increased by HCD, at both sampling times, only in dogs receiving dry commercial food before baseline (*p* = 0.002), while no differences emerged in dogs previously fed with foods already containing high water content, such as commercial wet foods or home-cooked diets. Concerning dog size, fecal moisture was significantly increased by HCD in both groups (small breeds: *p* = 0.038; large breeds: *p* = 0.017), with no differences between groups at baseline and after feeding with HCD (*p* > 0.05).

The fecal proportion of saturated fatty acids (SFAs) (+28%, *p* = 0.014; Table 4) and MCFAs (+162%, *p* = 0.012) increased from baseline after treatment with HCD + VCO. In particular, compared with baseline, the proportion of C6:0 increased after treatment with HCD (+79%, *p* = 0.005), while C10:0 and C12:0 increased after treatment with HCD + VCO (+183%, *p* = 0.023 and +600%, *p* = 0.001, respectively). In regard to other SFAs, compared to baseline, C14:0 and C22:0 increased both after treatment with HCD (C14:0 +74%, *p* = 0.005; C22:0 +58%, *p* = 0.008) and with HCD + VCO (C14:0 +142%, *p* < 0.001; C22:0 +33%, *p* = 0.037). Moreover, compared with baseline, the C16:0 proportion was higher in fecal samples collected after HCD + VCO treatment (+21%, *p* < 0.001), while C20:0 and C24:0 increased as the result of HCD treatment (+43%, *p* = 0.014 and +47%, *p* = 0.005, respectively). Compared with the baseline, the proportion of total MUFAs decreased both after treatment with HCD (−23%, *p* = 0.014) and with HCD + VCO (−29%, *p* = 0.014), mainly due to a significant reduction in C18:1cis-9 (−29%, *p* = 0.023 and −38%, *p* = 0.003 for HCD and HCD + VCO, respectively). Similarly, as a result of the reduction in C18:2n−6 following HCD + VCO treatment (−24%, *p* = 0.037), at the end of the study, the proportion of total PUFAs was lower than at baseline (−26%, *p* = 0.023). Regarding other PUFAs, the proportion of C18:3n−3 (+106%, *p* = 0.008) and C20:4n−6 (+56%, *p* = 0.019) were increased by HCD treatment.

**Figure 1:**
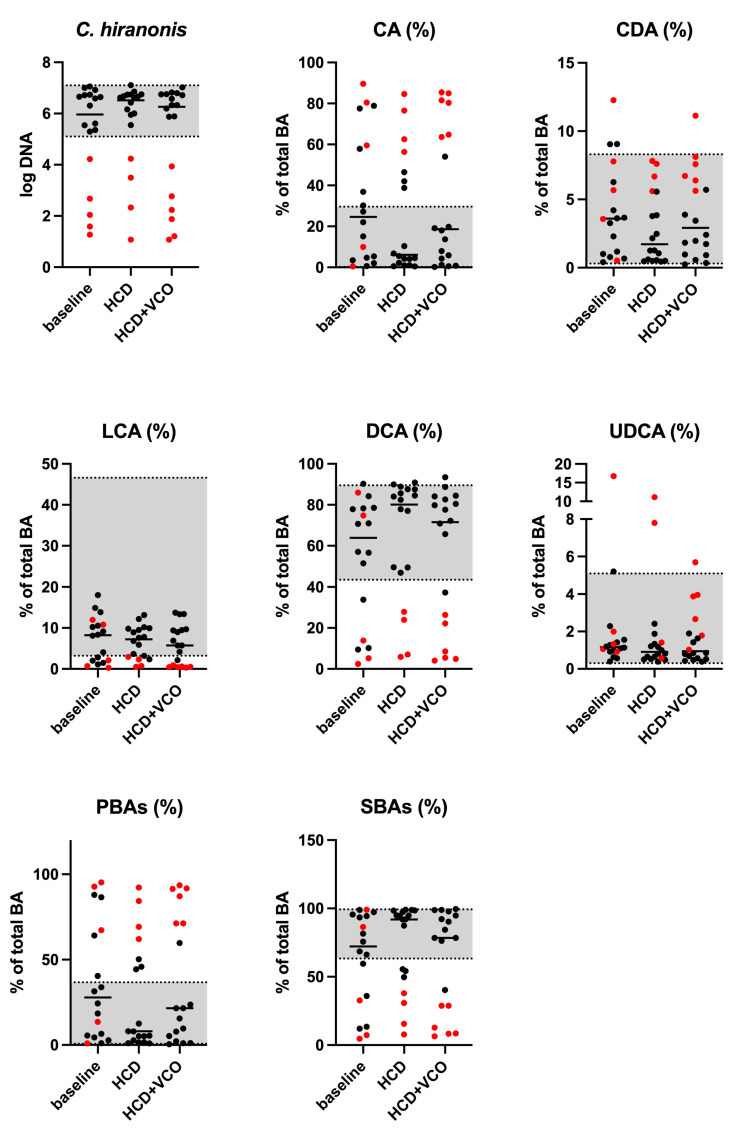
Dot plots showing the abundance of *C. hiranonis* (log DNA) and concentrations of the bile acids (BA, % of total) in canine fecal samples collected from dogs during the first visit (baseline), after receiving a home-cooked diet for 7 days (HCD), and after supplementation with virgin coconut oil (HCD + VCO). The black horizontal line represents the median. The grey shaded area corresponds to the internal reference intervals established for healthy dogs. The red dots represent dogs with low *C. hiranonis* abundance, while the black dots represent dogs with normal *C. hiranonis* abundance. Abbreviations: PBAs, primary bile acids [CA, cholic acid; CDA, chenodeoxycholic acid]; SBAs, secondary bile acids [LCAs, lithocholic acid; DCAs, deoxycholic acid; UDCAs, ursodeoxycholic acid].

Neither primary nor secondary fecal BA were influenced by dietary treatments (Figure 1).

Results concerning cholesterol, cholesterol intermediates (zoosterols), and phytosterols are reported in Table 5. Concerning zoosterols, fecal coprostanol concentration decreased following both dietary treatments (−27%, *p* = 0.007). Similarly, regarding fecal phytosterol concentrations, sitostanol decreased from baseline after treatment with HCD (−86%, *p* < 0.001), while campesterol decreased after treatment with HCD + VCO (−56%, *p* = 0.008).

However, fecal total measured sterols, which represent the sum of zoo- and phytosterols, were not influenced by dietary treatments, even though the phyto-/zoosterols ratio was lower by the end of the study (−61%, *p* = 0.005).

### 3.3. Fecal Microbial Analyses

**Figure 2:**
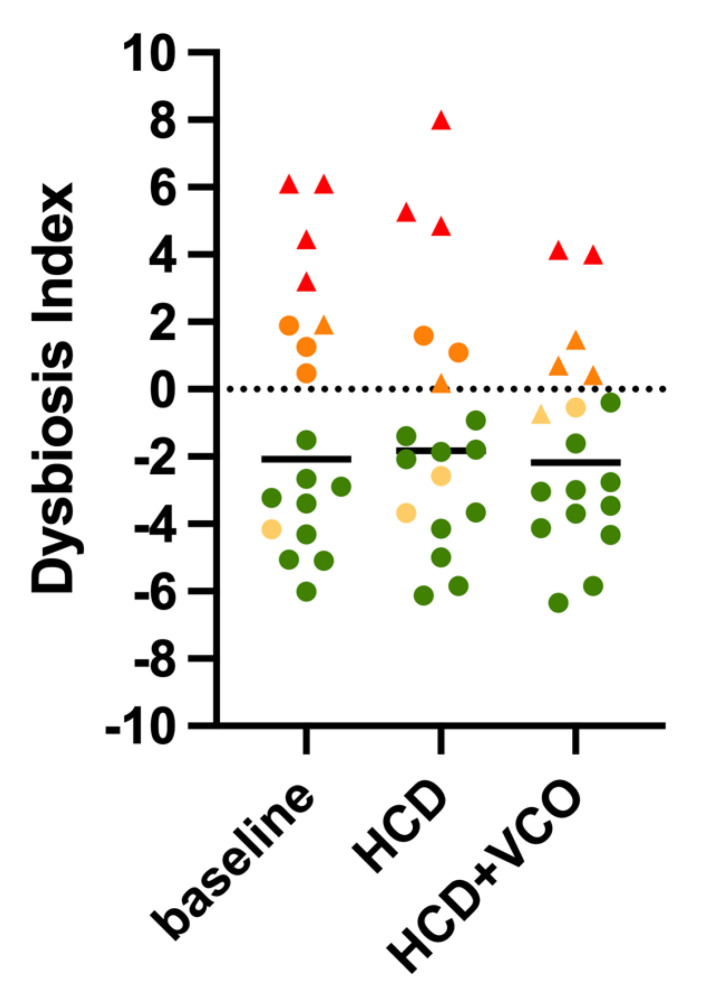
Dot plots showing the Dysbiosis Indexes (DIs) of canine fecal samples collected from dogs during the first visit (baseline), after receiving a home-cooked diet for 7 days (HCD), and after supplementation with virgin coconut oil (HCD + VCO). The black horizontal line represents the median. Interpretations for DI: <0 = normal; 0−2 = mildly increased; >2 = significantly increased. Graphic representations of the alterations (intended as deviation from the internal reference interval) of the selected bacterial populations, based on qPCR: green dots, normal; yellow dots, minor changes (defined as DI < 0 but some bacterial taxa outside their respective reference interval); orange dots, mild-to-moderate changes; red dots, significant changes. The triangles represent dogs with low *C. hiranonis* abundance.

Most of the microbial populations quantified in fecal samples were not affected by HCD or HCD + VCO (Table 6). Only the fecal abundances of total bacteria (*p* = 0.005) and *Fusobacterium* spp. (*p* = 0.028) were increased when dogs received HCD and HCD + VCO. The median DI was <0 and was not affected by dietary treatments (Table 6 and Figure 2), even though 8/18, 6/18, and 5/18 dogs showed mild-to-moderate or significant dysbiosis at baseline, after HCD treatment, and after HCD + VCO treatment, respectively. All CE dogs classified as clearly dysbiotic (DI > 2) had low *C. hiranonis* abundance (Figure 2).

## 4. Discussion

The objective of the present study was to investigate the effects of a home-cooked diet supplemented with VCO as a source of MCT on clinical scores and fecal microbiota and metabolome of CE dogs. The inclusion level of VCO provided in this trial was relatively low (10% of ME) to ensure a good acceptance of the diet by dogs. Indeed, the owners did not report food refusal problems. In this regard, a worsening of diet palatability has been previously described with a higher concentration of MCT supplemented in dogs (22–25% of ME) [25,26]. In accordance with our evidence, Beyen et al. [23] reported a total diet consumption in three dogs receiving a diet containing MCT at 11% of ME. In a recent study, Berk et al. [36] reported no differences in food intake in 19 healthy dogs when MCT oil was fed at 10% of ME in a short-term (5 day) palatability test.

All dogs responded positively to the home-cooked diet and were classified as food-responsive (FRE). Accordingly, in the literature, it is reported that FRE represents a very common condition, as it comprises approximately two-thirds of CE dogs [27,37]. In FRE dogs, an improvement in clinical signs is typically expected within a few days and usually no additional treatments are necessary [2,3]. In this study, clinical parameters such as CCECAI and FS showed a significant improvement throughout the study, and most of the dogs, by the end of the study, reached scores typically associated with healthy animals. However, at the end of the present study, higher CCECAI scores (5 or 6 were recorded, compared to baseline, in two out of the eighteen dogs, expressing, therefore, a moderate/severe disease still persistent after 37 days of dietary treatment. Moreover, these dogs were clearly dysbiotic (DI > 6) with a very low level of *C. hiranonis* (<2 log DNA) at baseline, and one showed suboptimal cobalamin concentration (the value was in the lower part of the reference range, <300 ng/L). Serum cobalamin concentrations of <350–400 ng/L have been associated with increased serum methylmalonic acid (MMA) concentrations [38]. MMA is known to accumulate as a result of the decreased intracellular availability of cobalamin and can impair the urea cycle [39]. However, MMA is currently not routinely performed in companion animals; therefore, cyanocobalamin supplementation is recommended when serum levels are <400 ng/L to avoid intracellular cobalamin deficiency in CE dogs, [39,40].

Concerning clinical scores, a beneficial effect attributable to VCO supplementation can be supposed only regarding FS, since this parameter was assessed before VCO supplementation (HCD) and at the end of the study (HCD + VCO). Conversely, CCECAI was assessed only at the beginning (baseline) and at the end of the study; consequently, it was not possible, regarding CCECAI, to differentiate the effect of HCD from that of VCO.

Unexpectedly, while FS displayed an improvement during the study, fecal water content slightly increased when dogs started to be fed with HCD. Water content is the main, but not the only, factor which influences fecal consistency; the fraction of fecal insoluble solids, its water holding capacity (WHC) properties, and the ratio of fecal water to fecal solids, are supposed to be other determinants of fecal firmness [41,42]. In this regard, the WHC of fecal insoluble solids has shown high variability in people with different dietary habits, showing that the ratio of fecal water to fecal solids depends mainly on diet composition [41]. For instance, potato fiber, the main dietary fiber source in HCD in this study, is known to possess a WHC capacity similar to psyllium (around 22 g water/g fiber source) [41,43,44]. Psyllium has been proven to improve fecal consistency in human diarrhea models, even though, at the same time, its ingestion was associated with an increase in fecal water content, probably due to its water-binding property [41,42]. The improvement of the FS and the higher fecal water content observed in this study might thus be explained by an increase in fecal viscosity depending on potato fiber intake, given the similar WHC capacity of psyllium and potato fiber. However, neither fecal viscosity nor the amount of fecal insoluble solid fraction was measured in this study. Consistent with our results, a linear decrease in fecal dry matter with no effect on FS was reported following an increase in potato fiber intake in healthy dogs [45]. Moreover, the high water content of HCD might have influenced fecal water excretion, as the increase in fecal moisture was more remarkable in dogs previously fed low-water foods, such as extruded dry kibbles. Factors other than diet, such as dog size and time of feces collection during the day, may cause variation in fecal consistency through the effect on transit time [46,47,48,49]. In this study, small- and large-sized dogs were included, and no impact of dog size on fecal water content was found. Unfortunately, the time of feces collection was not recorded during the day; therefore, we cannot exclude that this may have influenced fecal water content. To the best of the authors’ knowledge, to date, no studies have assessed differences in fecal characteristics in dogs fed home-cooked or commercial diets, but no differences in fecal water content in healthy dogs fed commercial wet or dry diets have been observed [50,51]. This latter finding is consistent with data showing that fluids are largely reabsorbed through the colonic mucosa in healthy dogs [52], but a reduction in intestinal absorptive capacity might be present in dogs with chronic gastrointestinal disorders, due to a more rapid colonic transit time [50,53].

The chemical analysis highlighted some interesting modulations on fecal fatty acid profile during the study, presumably induced by dietary treatments. In particular, an increase in the fecal proportion of MCFAs (C10:0 and C12:0) and C14:0, together with a reduction in C18:1c9 (MUFA), was observed as a consequence of VCO supplementation. The VCO is a lipidic source containing more than 92% SFAs (MCFAs, in particular), as previously reported [18]. On the other hand, the increase in the fecal proportions of C16:0, C18:3n3, and C20:4−n6 following HCD consumption can be associated with the fatty acid profile of horse meat [54,55]. In humans, SFAs are generally considered to be hypercholesterolemic, whereas MUFAs and PUFAs are considered hypocholesterolemic [18]. In this study, VCO consumption increased total fecal SFAs without affecting serum cholesterol levels in CE dogs.

A digestibility assessment was outside of the scope of this study, but MCFAs are considered to be almost completely absorbed by the intestinal mucosa as free fatty acids, without requiring the action of bile salts and pancreatic lipase [56]. However, this absorptive mechanism is still controversial in dogs, since MCFAs have been found to be absorbed in canine lymph [57]. Among MCFAs, C8:0 and C10:0 have short half-lives and rapidly enter hepatic mitochondria; therefore, they are presumably not incorporated into chylomicrons [58]. Partially in agreement with this evidence, in the present study, an increase in the fecal proportion of C10:0, but not of C8:0, was exerted by VCO supplementation. Therefore, along with our results, an impaired intestinal passive absorption of MCFAs with carbon chains longer than eight carbons might be suspected in CE dogs. In this regard, because VCO contains prevalently C12:0 (and C14:0), purified MCT oils (high in C8:0 and C10:0), rather than VCO, could represent a preferable dietary MCFA source to be used in CE dogs [59]. Moreover, investigations are needed to understand if MCFA absorption takes place in dogs via the portal venous system rather than via the lymphatic system, as has been observed in other animal species.

The overall fat content measured in fecal samples collected after the beginning of HCD treatment displayed a reduction in fat excretion compared to the baseline. The availability of data on fecal fat content is limited in dogs [60,61,62], and a threshold value for excessive fecal fat excretion, identifiable as steatorrhea, has been not established so far. In human beings, an excretion of 4–7 g/fecal lipid/day (or fat <5% fecal wet weight) is considered normal, while a fecal fat content >9.5% is considered risky for steatorrhea [63,64]. In a study with healthy dogs fed a dry extruded diet, fecal fat loss depended on dog size, ranging from 1.2 to 14 g/fecal lipid/day for small to large size dogs, respectively [61]. In another study, fecal fat excretion did not change in response to a progressive increase in dietary fat content (from 15 to 50 g/lipids/day) in healthy dogs, while a linear increase in fecal fat loss was observed when those dogs were deprived of the gallbladder [60]. More recently, fecal fat loss was evaluated in healthy Beagle dogs in relation to different dietary treatments that consisted of a basal diet with increasing levels of poultry fat [62]. In that study, when dogs were fed a diet containing 7.8% fat on a DM basis, a value close to that of the HCD diet used in the present study (7.9% fat on a DM basis), the fat content of feces was twofold higher than in our study (5% vs. 2.6% on a DM basis). This discrepancy could be explained by the composition of the different diets; in fact, differences in fat and fiber sources and levels of inclusion can result in considerable variation in fecal fat losses in dogs and pigs [65,66]. Interestingly, in the cited study with Beagle dogs [62], fecal fat content was significantly higher when dogs were fed diets with fat content equal to or higher than 13% on a DM basis, compared to a 7.8% fat diet. The fat content of the diets that were fed to dogs before the HCD diet was not investigated in the present study, but a fat content higher than that of HCD could be supposed for dog adult maintenance diets; therefore, our results might mirror the study by Marx et al. [62]. Other authors [67] observed no changes in the fecal fat content of dogs as dietary fat levels increased, but a lower fecal output was registered in that case, and this might have accounted for the lack of difference in the fecal fat content. The total fecal output was not weighed in the present study; thus, speculation about the reduction in indigestible fecal matter content secondary to HCD, as well as an improvement of fat digestibility in CE dogs fed HCD, cannot be confirmed, even though the latter would support the clinical improvement also highlighted by CCECAI. However, in addition to unabsorbed dietary fat, fecal fat also comprised endogenous fat loss, which are molecules originating from bile salts, cholesterol, and the structural lipids of mucosal cells that pass through the intestinal tract to be finally excreted with feces [68].

In this study, fecal sterol composition was affected by dietary treatments. In fact, the introduction of HCD, a diet rich in proteins from an animal source, horse meat, lead to a reduction in phytosterols and the phytosterol-to-zoosterol ratio in dog feces. On the other hand, a reduction in fecal campesterol concentration was observed secondary to VCO supplementation; campesterol is commonly found in the highest amounts in vegetable oils, with those extracted from seeds having around five-fold higher amounts of campesterol than VCO [69].

Results from recent studies in dogs suggest a decreased concentration of β-sitosterol and sitostanol in CE dogs compared to healthy subjects, with no difference regarding zoosterols [33,70]. Compared to cholesterol, phytosterols are bioactive compounds poorly absorbed by the intestine (2–3% vs. 30–60% absorption rate for phytosterols and cholesterol, respectively [71]); therefore, fecal phytosterol concentration better reflects dietary composition than zoosterols. In this study, total phyto- and zoosterol fecal concentrations were similar to values observed in CE Yorkshire Terrier dogs [33].

Previous studies have demonstrated alterations in intestinal BA metabolism in CE dogs [72,73]. In the present study, concentrations of fecal BA, cholesterol, and its intermediates were not affected by HCD and VCO. However, a numerical decrease in the fecal excretion of primary bile acids (mainly due to a decrease in cholic acid) occurred when HCD was fed to dogs, but the high variability of fecal BA concentrations among CE dogs might have weakened the statistical power. Differences in the fecal BA concentrations of healthy and CE Yorkshire Terrier dogs have recently been described by Galler et al. [33]. Moreover, Pezzali et al. [74] hypothesized differences in BA concentration among different dog breeds. Studies on alterations of the intestinal BA metabolism have been suggested to be a promising target for fecal analysis because of their important role in host metabolism and their relationship with gut bacteria [75]. In particular, the intestinal microbiota affects bile acid metabolism through deconjugation and dihydroxylation of primary BA to form secondary BA [73]. In dogs, *C. hiranonis* represents the main bacterial converter of primary to secondary BA [76]. Since the abundance of this bacterial species is often depleted in CE dogs or, in general, in dogs presenting with intestinal dysbiosis, in these situations an increase in primary BA (potentially the cause of secretory BA diarrhea) and a decrease in secondary BA has been often observed [75]. In this study, fecal concentrations of *C. hiranonis* were not significantly affected by HCD or VCO supplementation, and this result is in accordance with the lack of changes in BA metabolism. In fact, the results from the present study confirmed the inability to convert primary to secondary BA in dogs with low *C. hiranonis* abundance.

The CE dogs involved in this study were not all affected by gut dysbiosis since the median DI was lower than 0, which is considered normal [34]. Moreover, DI was not significantly affected by dietary treatments, and all dogs classified as clearly dysbiotic had low *C. hiranonis* abundance at each sampling time. Contrary to the clinical score (CCECAI), which includes symptoms such as vomiting and diarrhea that might improve rapidly in response to dietary treatment, the DI rather reflects the changes on a mucosal level which might take longer to recover.

The abundance of total bacteria, as well as *Fusobacterium* spp., increased as a result of feeding with the HCD. Fusobacterium is a co-dominant phylum of fecal microbiota in healthy dogs, and its abundance is associated with intestinal health [77], with a subset of CE dogs showing a lower abundance of *Fusobacterium* spp. compared to healthy dogs [78]. Some bacterial species belonging to Fusobacteria are known to produce butyrate from dietary protein [77], and its growth in dogs is stimulated by high-protein diets, such as raw meat-based diets [79,80]. Among short-chain fatty acids (SCFAs), butyrate is the most important energy source for colonocytes and has shown local and systemic anti-inflammatory effects, as well as increased expression of immunosuppressive cytokines in human ulcerative colitis [81,82]. Even though the fecal abundance of *Fusobacterium* spp. in CE dogs in this study was within the reference range previously attributed to healthy dogs [77], the HCD significantly increased the fecal concentration of this bacterial genus only a few days after starting the dietary treatment. Conversely, the fecal depletion of *Fusobacterium* spp. caused by severe dysbiosis or antibiotics treatments would probably take longer to restore, as shown in a previous study where Fusobacteria abundance remained depleted in healthy dogs treated with metronidazole, even 4 weeks after metronidazole discontinuation [83]. In this study, we did not evaluate the effects exerted by HCD or VCO on fecal SCFA and other postbiotics, even though future nutritional interventions involving substances released by or produced through the metabolic activity of intestinal bacteria may significantly benefit the host in disease conditions linked to the microbiome, such as chronic intestinal inflammation [84].

The study has some limitations that should be considered. Firstly, the dogs used in this study were client-owned and were fed different habitual diets that could have resulted in a potential confounding factor in the assessment of the effects exerted by the HCD treatment on the fecal microbiota, sterol, and fatty acid profile of CE dogs. In addition, HCD was fed to CE dogs for only 7 days before adding VCO; therefore, it cannot be differentiated if the improvement in FS observed at the end of the study was associated with VCO supplementation or a delayed effect associated with HCD. Finally, we cannot exclude that an improvement in parameters other than clinical signs, such as the DI, would have been seen if the dietary treatment was tested long-term.

## 5. Conclusions

In the present study, a home-cooked diet, with and without VCO supplementation at 10% ME, was tested as a dietary treatment on 18 dogs with CE. All dogs responded positively to dietary treatments, which improving their clinical condition, and were classified as dietary responders. The HCD reduced fecal fat excretion, and VCO supplementation was effective in improving fecal consistency. This study highlighted that the levels and sources of dietary fat significantly affect the fecal fatty acid and sterol profiles of CE dogs. Moreover, the fecal abundance of bacterial populations, which are linked to high animal protein diets, was increased by the HCD.

The VCO supplementation failed to exert any significant modulatory effect on fecal microbiota and resulted in higher fecal proportions of certain MCFAs. Further research is warranted to better clarify the absorptive mechanism of MCFAs in dogs and to determine whether VCO increases the fecal proportion of MCFAs in healthy dogs as well.

## Figures and Tables

**Table 1:** Ingredients and chemical composition of the experimental diets fed to CE dogs.

Ingredients (g/100 g)	HCD	HCD + VCO
Potatoes	59.5	59.5
Horse meat	29.7	29.7
Zucchini	8.5	8.5
Sunflower oil	1.5	0.5
Linseed oil	0.2	0.2
VCO	/	1.0 *
Minerals and vitamins supplement	0.6	0.6
Chemical composition (%)		
Moisture	80.47	80.42
Dry matter (DM)	19.53	19.58
% on DM basis		
Crude protein	39.2	39.0
Crude fat	7.9	8.1
Starch	17.97	18.12
Crude fiber	1.30	1.47
Ash	6.29	6.27

* VCO supplementation provided 10% of metabolizable energy. HCD, home-cooked diet; VCO, virgin coconut oil.

**Table 2:** Fatty acids composition (% of total fatty acids) of the experimental diets and the virgin coconut oil used in the study.

Fatty Acid, %	HCD	HCD + VCO	VCO
C6:0	1.30	0.42	0.67
C8:0	0.43	6.21	10.37
C10:0	0.14	3.72	6.11
C12:0	0.16	26.42	44.36
C14:0	2.87	14.16	21.24
C16:0	31.91	17.06	7.86
C18:0	8.16	4.65	3.03
C20:0	0.42	0.16	0.08
C22:0	1.19	0.24	n.d.
C24:0	0.42	0.12	n.d.
Total MCFAs	2.03	36.77	61.51
Total SFAs	47.00	73.16	93.72
C16:1cis-9	4.60	2.76	n.d.
C18:1cis-9	44.46	21.99	5.28
Total MUFAs	49.06	24.75	5.28
C18:2n−6	3.30	2.05	1.00
C18:3n−3	0.50	n.d.	n.d.
C20:4n−6	0.14	0.04	n.d.
Total PUFAs	3.94	2.09	1.00

HCD, home-cooked diet; MCFAs, medium-chain fatty acids; SFAs, saturated fatty acids; MUFAs, monounsaturated fatty acids; PUFAs, polyunsaturated fatty acids; VCO, virgin coconut oil; n.d., not detected.

**Table 3:** Clinical scores, body weight, and selected blood parameters assessed in dogs during the first visit (baseline) and after the addition of virgin coconut oil to the home-cooked diet (HCD + VCO). Data are expressed as median and range.

	Baseline	HCD + VCO	*p*-Value
Clinical parameters			
BW (kg)	15.8	15.6	0.711
	5.3–40.0	4.6–39.7	
BCS (9 points scale)	4.5	4.5	0.717
	2–6	3–5	
CCECAI	3	1	0.001
	1–10	0–6	
Selected serum parameters			
Cobalamin (ng/L)	286	/	
RI [250–730]	150–940		
Cholesterol (mg/dL)	199	176	0.396
RI [123–345]	120–278	119–273	
Triglycerides (mg/dL)	43	38	0.807
RI [30–120]	18–88	27–175	
Albumin (g/dL)	3.14	3.10	0.659
RI [2.75–3.85]	1.85–3.53	2.1–3.7	
Total protein (g/dL)	6.42	6.53	0.632
RI [5.60–7.30]	4.34–7.1	5.52–6.90	

BW, body weight; BCS, body condition score; CCECAI, canine chronic enteropathy clinical activity index; RI, reference intervals.

**Table 4:** Chemical parameters measured in canine fecal samples collected in dogs during the first visit (baseline), after receiving a home-cooked diet for 7 days (HCD), and after supplementation with virgin coconut oil (HCD + VCO). Data are expressed as median and range.

	Baseline	HCD	HCD + VCO	*p*-Value
Water content (%)	71.5 a	77.5 b	76.7 b	0.001
	57.8–80.4	73.7–85.4	72–81.7	
Fat content (%, on a DM basis)	3.50 a	2.60 b	2.75 ab	0.034
	1.3–8.9	1.1–6.6	1.3–4.8	
Fatty acids (% of total FA)				
C6:0	0.14 a	0.25 b	0.22 ab	0.006
	0.06–0.84	0.11–0.60	0.11–1.92	
C8:0	0.13	0.33	0.21	0.065
	0.02–0.52	0.04–1.52	0.06–2.32	
C10:0	0.06 a	0.12 ab	0.17 b	0.015
	0.009–0.33	0.016–1.07	0.05–1.93	
C12:0	0.09 a	0.23 a	0.63 b	0.001
	0.016–2.4	0.031–3.97	0.081–8.21	
C14:0	1.35 a	2.36 b	3.27 b	<0.001
	0.64–5.1	0.39–5.8	1.27–15.3	
C16:0	28 a	31 a	34 b	<0.001
	14–35.2	16.3–37.9	23.9–47.9	
C18:0	22.0	23.0	23.8	0.513
	4.64–41.8	14.3–33.1	13.5–43.9	
C20:0	0.94 a	1.34 b	1.23 ab	0.012
	0.43–2.41	0.71–2.22	0.66–1.90	
C22:0	1.01 a	1.60 b	1.34 b	0.006
	0.4–21.9	0.54–7.30	0.62–4.95	
C24:0	1.30 a	1.91 b	1.65 ab	0.005
	0.64–7.04	0.75–3.08	0.69–3.06	
Total MCFAs	0.50 a	1.14 ab	1.31 b	0.012
	0.11–3.14	0.30–5.44	0.80–9.17	
Total SFAs	55.2 a	65.5 ab	70.8 b	0.014
	24–79	39–75	49–80	
C16:1cis-9	1.51	2.33	2.37	0.060
	0.21–4.52	0.23–3.76	0.66–3.33	
C18:1cis-9	23.3 a	16.6 b	14.5 b	0.002
	8.35–33.4	3.61–46.5	8.29–27.5	
Total MUFAs	24.6 a	19.0 b	17.5 b	0.002
	9.87–35.3	7.37–48.7	9.39–29.7	
C18:2n−6	17.0 a	15.5 ab	12.9 b	0.030
	5.92–52.1	6.05–31.0	8.42–23.3	
C18:3n−3	0.36 a	0.74 b	0.51 ab	0.009
	0.049–4.43	0.042–6.30	0.045–3.82	
C20:4n−6	0.41 a	0.64 b	0.60 ab	0.018
	0.075–1.19	0.30–1.56	0.28–1.43	
Total PUFAs	19.8 a	17.1 ab	14.6 b	0.024
	8.62–52.3	6.91–32.0	9.09–24.3	

MCFAs, medium-chain fatty acids; SFAs, saturated fatty acids; MUFAs, monounsaturated fatty acids; PUFAs, polyunsaturated fatty acids. Within the same row, medians without a common letter differ (*p* < 0.05).

**Table 5:** Concentrations of sterols (µg/mg of dry feces) in canine fecal samples dogs during the first visit (baseline), after receiving a home-cooked diet for 7 days (HCD), and after supplementation with virgin coconut oil (HCD + VCO). Data are expressed as median and range.

Sterols	Baseline	HCD	HCD + VCO	*p*-Value
Coprostanol	0.056 a	0.041 b	0.041 b	0.007
RI [0.04–0.19]	0.031–0.12	0.031–0.058	0.035–0.055	
Cholesterol	5.79	6.26	6.48	0.837
RI [0.81–8.83]	1.83–14.6	2.57–14.4	3.05–14.3	
Cholestanol	0.19	0.21	0.24	0.137
RI [0.05–0.42]	0.048–0.32	0.14–0.43	0.12–0.58	
Lathosterol	0.034	0.039	0.045	0.211
RI [0.02–0.06]	0.024–0.07	0.024–0.22	0.025–0.11	
Total Zoosterols	3.77	5.54	5.06	0.179
RI [0.93–9.24]	1.94–14.9	2.79–14.8	3.28–14.6	
Brassicasterol	0.055	0.047	0.046	0.056
RI [0.04–0.23]	0.04–0.16	0.04–0.06	0.038–0.16	
Campesterol	0.95 a	0.58 ab	0.42 b	0.011
RI [0.27–2.27]	0.32–2.95	0.15–1.41	0.13–1.24	
Stigmasterol	0.52	0.39	0.39	0.329
RI [0.08–1.06]	0.15–1.28	0.14–1.01	0.12–0.65	
Fusosterol	0.08	0.11	0.10	0.301
RI [0.03–0.22]	0.03–0.32	0.06–0.16	0.06–0.26	
β-sitosterol	1.84	1.75	1.28	0.411
RI [0.37–4.28]	0.91–7.29	0.40–6.64	0.41–3.81	
Sitostanol	0.072 a	0.01 b	0.01 b	<0.001
RI [0.01–0.74]	0.031–0.41	0.01–0.12	0.10–0.11	
Total Phytosterols	3.67	2.88	2.19	0.179
RI [0.82–7.71]	1.67–12.1	0.79–9.24	0.80–5.99	
Total Phyto- to Zoosterols	0.93 a	0.42 ab	0.36 b	0.006
RI [0.20–3.54]	0.15–2.69	0.07–1.70	0.08–1.13	
Total sterols	8.11	9.15	8.51	0.946
RI [2.31–20]	4.18–27	3.62–18	4.31–16	

Within the same row, medians without a common letter differ (*p* < 0.05). RI, reference intervals.

**Table 6:** Fecal abundance of selected bacterial populations based on qPCR, in canine fecal samples collected from dogs during the first visit (baseline), after receiving a home-cooked diet for 7 days (HCD), and after supplementation with virgin coconut oil (HCD + VCO). Data are expressed as median and range.

Bacterial Populations (Log DNA)	Baseline	HCD	HCD + VCO	*p*-Value
Universal bacteria	11.1 a	11.2 b	11.3 b	0.005
	10.7–11.4	10.9–11.6	11.0–11.6	
*Faecalibacterium* spp.	5.58	4.77	4.83	0.556
RI [3.4–8.0]	3.45–6.83	2.76–7.26	2.82–7.25	
*Turicibacter* spp.	7.37	7.35	7.0	0.854
RI [4.6–8.1]	5.74–8.12	6.07–8.24	5.79–8.67	
*Streptococcus* spp.	5.07	5.54	4.71	0.198
RI [1.9–8.0]	3.04–8.93	3.33–8.87	2.75–7.33	
*E. coli*	7.26	6.91	7.24	0.945
RI [0.9–8.0]	4.42–8.84	3.60–8.47	4.22–7.89	
*Blautia* spp.	10.7	10.7	10.6	0.385
RI [9.5–11]	7.07–11.1	9.73–11.1	10.0–11.3	
*Fusobacterium* spp.	9.27 a	9.80 b	9.85 b	0.028
RI [7.0–10.3]	7.37–10.0	7.80–10.5	7.50–10.4	
*C. hiranonis*	5.96	6.52	6.26	0.379
RI [5.1–7.1]	1.27–7.06	1.08–7.11	1.08–7.02	
Dysbiosis Index	−2.08	−1.83	−2.18	0.680

RI, internal reference intervals established for healthy dogs. Within the same row, medians without a common letter differ (*p* < 0.05).

## Data Availability

The data presented in this study are available in this article.
